# Th Inducing POZ-Kruppel Factor (ThPOK) Is a Key Regulator of the Immune Response since the Early Steps of Colorectal Carcinogenesis

**DOI:** 10.1371/journal.pone.0054488

**Published:** 2013-01-17

**Authors:** Francesco Mariani, Paola Sena, Monica Pedroni, Piero Benatti, Paola Manni, Carmela Di Gregorio, Antonio Manenti, Carla Palumbo, Maurizio Ponz de Leon, Luca Roncucci

**Affiliations:** 1 Department of Internal Medicine, University of Modena and Reggio Emilia, Modena, Italy; 2 Department of Morphological Sciences, University of Modena and Reggio Emilia, Modena, Italy; 3 Department of Servizi Diagnostici di Laboratorio e Medicina Legale, University of Modena and Reggio Emilia, Modena, Italy; 4 Department of Surgery, University of Modena and Reggio Emilia, Modena, Italy; Mie University Graduate School of Medicine, Japan

## Abstract

We purposed to evaluate the role of Th inducing POZ-Kruppel Factor (ThPOK), a transcriptional regulator of T cell fate, in tumour-induced immune system plasticity in colorectal carcinogenesis. The amounts of CD4+, CD8+ and CD56+ and ThPOK+ cells infiltrate in normal colorectal mucosa (NM), in dysplastic aberrant crypt foci (microadenomas, MA), the earliest detectable lesions in colorectal carcinogenesis, and in colorectal carcinomas (CRC), were measured, and the colocalization of ThPOK with the above-mentioned markers of immune cells was evaluated using confocal microscopy. Interestingly, ThPOK showed a prominent increase since MA. A strong colocalization of ThPOK with CD4 both in NM and in MA was observed, weaker in carcinomas. Surprisingly, there was a peak in the colocalization levels of ThPOK with CD8 in MA, which was evident, although to a lesser extent, in carcinomas, too. In conclusion, according to the data of the present study, ThPOK may be considered a central regulator of the earliest events in the immune system during colorectal cancer development, decreasing the immune response against cancer cells.

## Introduction

Solid tumours are commonly infiltrated by several immune cells [1]–[3]. In cancer, immune cells play conflicting roles with potential capability either in eliminating or promoting malignancy. In contrast to infiltration of cells responsible for chronic inflammation, the presence of high numbers of lymphocytes, especially T cells, has been reported to be an indicator of good prognosis in many types of cancer [4]–[7]. However, even if the abundance of tumour-infiltrating T-cells has been associated with improved clinical outcome, in some types of cancer, including the colorectal ones, the influence of immune cells on the prognosis is still a matter of debate. Although the exact mechanism remains uncertain, the adaptive immune system may play an important role in suppressing tumour progression [8]. Tumour-infiltrating T-cells may be suggestive of the host immune response to the tumour and represent attractive targets for immunotherapy [9].

Both CD4+ and CD8+ T cells are required for effective tumour cell elimination. It is well recognized that cytotoxic T lymphocytes (CD8+ T cells) are crucial components of antitumour immunity, since activated CD8+ T cells can directly kill tumour cells by the release of granules including lytic components such as perforin and enzymatic proteases (like granzyme B, GZMB) [10]–[12]. In a recent investigation it was reported that the degree of infiltration with CD8+ T cells is inversely correlated to the tumour stage and the early signs of metastasis [13].

CD4+ T lymphocytes play a central role in orchestrating both onset and maintenance of the adaptive immune response. Some studies have suggested that a high CD8+/CD4+ T-cell ratio as well as a high frequency of activated CD8+ T cells in colon cancer are associated with the presence of an activated anticancer immune reaction [14]. Furthermore, tumour tissue selective infiltration of CD4+ T helper cells in colorectal cancer has been demonstrated [15]. Increased infiltration of CD4+ T cells in tumours may also be due to a greatly enhanced number of Foxp3+ regulatory T cells, that would explain the insufficiency of the immune system to adequately attack primary tumours [16]. However, the function and phenotype of tumour infiltrating CD4+ T cells in colorectal cancer has not been yet characterized.

Natural killer (NK) cells and Natural killer T cells (NKT) are CD56+ innate lymphocytes which have different biological functions including the ability to recognize and kill a variety of tumour cells before the antigen sensitization or clonal expansion [17]–[20]. Recent studies indicate that these cells are scarce in CRC tissue since the early stages, compared to nonmalignant colonic tissue, and that a decreased number of CD56+ cells in patients with CRC is associated with an increased frequency of cancer recurrence [21]–[24].

It remains important, therefore, to better understand how tumours can evade immune-mediated attack once established. The strategies to escape anti-tumor immune responses include the limited priming or differentiation of antitumour T cells and the role of tumour microenvironment to prevent infiltration or activation of effector phase functions.

The Zbtb7b gene (referred to as ThPOK, T helper-inducing POZ–Kruppel-like factor) is a transcriptional regulator, which is necessary and sufficient to induce the commitment of the helper lineage rather than the cytotoxic one in the T-cell subsets. ThPOK is necessary for mediating CD4+ commitment and preventing CD8+ commitment. Important is the key function of Zbtb7b in preventing the expression of cytotoxic differentiation markers like perforin and CD103 granzyme B, and the transcription factors RUNX3 and Eomes [25]–[27]. It has been reported that ThPOK expression into CD8+ T cells, in which normally it is not expressed, results in the loss of some CD8+ T cell characteristics like the expression of CD8 receptor and cytotoxic effector genes, and in the up-regulation of genes typically expressed in helper differentiation, including enhanced IL-2 production, although not of CD4 itself [28], [29].

Given the crucial role of ThPOK in cell fate determination of the helper lineage, we evaluated ThPOK expression and quantification along colorectal cancer development since its early steps, including dysplastic aberrant crypt foci, referred to as microadenomas [30]. The results of the present study suggest that ThPOK can play an important role in controlling adaptive immunity against colorectal tumours, since the early phases of neoplastic transformation.

## Materials and Methods

### Ethics Statement

All patients enrolled in this study, which underwent colonoscopy or surgical resection for colorectal cancer at the University Hospital of Modena, were asked to give an informed written consent to this study protocol, which was specifically approved by the Comitato Etico Provinciale di Modena.

### Study Population

Sixty samples of normal colorectal mucosa (NM) were collected from 20 patients during colonoscopy, 3 samples for each patient. One sample for each patient was fixed in formalin and embedded in paraffin for histology and RNA extraction; the others were frozen at −80°C. All patients had normal colonoscopy. Thirty microadenomas (MA) were also identified in 11 patients, and removed after operation for colorectal cancer on surgical specimens, after staining of the mucosa with a 0.1% methylene-blue solution in saline, and observation under a dissecting microscope [30]. The average multiplicity of the MA examined is 79 (range 30–120), and all showed low grade dysplasia. One MA for each patient was fixed in formalin and embedded in paraffin, the others were frozen at −80°C. Finally, 60 samples of colorectal cancer (CRC) were collected from 20 patients operated on for colon or rectal cancer, a sample for each patient was fixed in formalin and the other two were frozen at −80°C, as described above.

### Western Blot Analysis

One sample frozen at −80°C for each subject was used for western blot analysis. Whole cell lysates were obtained from 20 samples of NM, 10 MA, and 20 CRC, extracted with hypotonic buffer (50 mM Tris-Cl, pH 7.8, containing 1% Nonidet P40, 140 mM NaCl, 0.1% SDS, 0.1% Na deoxycholate, 1 mM Na_3_VO_4_, and freshly added protease inhibitor cocktail). Lysates were then cleared by centrifugation for 15 min in a refrigerated centrifuge, max speed, and immediately boiled in SDS sample buffer. Forty µg of protein extract from each sample (NM, MA, and CRC) were electrophoresed on SDS-PAGE and transferred to nitrocellulose membranes. The membranes wereblocked with 3% dry milk and 2% BSA in PBS-T and was incubated with the following antibodies, diluted 1∶1000 overnight at 4°C under agitation: rabbit anti-zbtb7b (Sigma), mouse anti-CD4, mouse anti-CD8, mouse anti-CD56 (Dako). After washing, the membranes were incubated with secondary HPR-conjugated goat anti-rabbit IgG antibody or goat anti-mouse IgG antibody (1∶10000) for 30 min at room temperature. Immunoreactive proteins were detected with ECL (Amersham). Anti-mouse-α-actin (Sigma) was used as loading control. Densitometry analysis was performed using a KODAK (Rochester, NY) Image Station 440 cf system, and semiquantitative analysis was performed with NIH Image J software. For each sample and for each marker, the band intensities were normalized to *α*-actin and results are expressed as the normalized treatment to control ratio.

### Evaluation of Immunofluorescence by Confocal Microscopy

One sample frozen at −80°C for each subject was used for immunofluorescence analysis to evaluate the expression of ThPOK, CD4, CD8, CD-56, GZMB, RUNX3, and Foxp3, proteins. Twenty samples of NM, 10 MA, and 20 CRC were fixed in 4% paraformaldehyde in PBS, cryoprotected in 15% sucrose in PBS, and frozen in iso-pentane cooled in liquid nitrogen. Horizontal cryosections of the samples were cut (10 µm thick), and haematoxylin and eosin staining was performed on sections to control tissue integrity and histology. After a treatment with 3% BSA in PBS for 30 min at room temperature, the cryostatic sections were incubated with the primary antibodies (rabbit anti-zbtb7b (Sigma), mouse anti-CD4, mouse anti-CD8, mouse anti-CD56 (Dako), goat anti-Foxp3, goat anti-RUNX3 or anti granzyme B (anti-GZMB) (Santa Cruz); diluted 1∶25 in PBS containing 3% BSA for 1 h at room temperature. After washing in PBS, the samples were incubated for 1 h at room temperature with the secondary antibodies diluted 1∶20 in PBS containing 3% BSA (sheep anti-mouse FITC conjugated, goat anti-rabbit TRITC conjugated; sheep anti-goat CF^TM^64 conjugated (SIGMA)). After washing in PBS and in H_2_O, the samples were counterstained with 1µg/ml DAPI in H_2_O and then mounted with anti-fading medium (0.21 M DABCO and 90% glycerol in 0.02 M Tris, pH 8.0). Negative control samples were not incubated with the primary antibody. The confocal imaging was performed on a Leica TCS SP2 AOBS confocal laser scanning microscope.

Excitation and detection of the samples were carried out in sequential mode to avoid overlapping of signals. Sections were scanned with laser intensity, confocal aperture, gain and black-level setting kept constant for all samples. Optical sections were obtained at increments of 0.3 µm in the z-axis and were digitized with a scanning mode format of 512 x 512 or 1024 x 1024 pixels and 256 grey levels. The confocal serial sections were processed with the Leica LCS software to obtain three-dimensional projections. Image rendering was performed by adobe Photoshop software. The original green fluorescent confocal images were converted to grey-scale and median filtering was performed. An intensity value ranging from 0 (black) to 255 (white) was assigned to each pixel.

Background fluorescence was subtracted and immunofluorescence intensity (IF) was calculated as the average for each selected area. The fluorescence intensity at the selected areas, linearly correlated with the number of pixels, was quantitatively analysed using the standard imaging analysis software of an NIS-Elements system. To each sample was assigned a code number and the score, referred to as ImmunoFluorescence Intensity Score (IFIS), was determined by an observer who was blind to tissue groups during analysis [31], [32].

### RNA Isolation and Quantitative Real Time-PCR

One sample formalin fixed and paraffin embedded for each patient was used for histological evaluation and mRNA extraction and quantification. Total RNA was isolated from formalin-fixed, paraffin-embedded samples (the last 20 samples of NM, 10 MA, and 20 CRC). Total RNA was extracted from 10 µm sections, using High Pure RNA Paraffin Kit (Roche Diagnostics S.p.A., Milan, Italy) according to the manufacturer’s instructions; this was followed by DNase treatment and removal of contaminating DNA from the RNA. RNA content was determined by spectrophotometry and first-strand cDNA was synthesized from 2 µg of total RNA with Superscript VILO cDNA synthesis kit (Invitrogen S.r.l., Milan, Italy) according to the manufacturer’s instructions.

Real-time PCR was done on the iCycler iQReal-Time Detection System (Bio-Rad) using Go-taq qPCR Master Mix (Promega, Milan, Italy) according to detailed manufacturer’s protocols. The iQReal-Time Detection System software generated a standard curve from 10-fold serial cDNA dilutions, and the threshold cycle was normalized for each standard curve. To confirm amplification of a specific product, melting curve analysis was performed and PCR products were directly visualized on 2% low-melting agarose gels. The copy numbers for all samples were normalized with the data obtained from GAPDH, which was used as a control. Data were expressed as fold induction in pathological tissue referred to normal mucosa (ΔCt Method Using a Reference Gene or Livak Method) [33].

Specific primers for zbtb7b were designed using Primer3 software (Perkin-Elmer Applied Biosystems). Total gene specificity of the nucleotide sequence chose for primer was confirmed by results of BLAST searches (GenBank database sequences). Specific primer pairs were as follows: zbtb7b, forward 5′-tgagaggagaagatggggag-3′ and reverse 5′-cggatggtgaggtcacatag-3′; GAPDH, forward 5′-agccacatcgctcagacac-3′ and reverse 5′-gcccaatacgaccaaatcc-3′. The cycling conditions were established as follows: single cycle at 95°C for 2 minutes, 40 cycles at 95°C for 15 seconds, and at 60°C for 60 seconds.

### Colocalization Analysis

To examine the cellular localization of ThPOK within CD4+, CD8+, CD56+ cells, multiple immunofluorescence staining of rabbit anti-zbtb7b antibody(Sigma) with mouse anti-CD4, mouse anti-CD8, mouse anti-CD56 (Dako), goat anti-Foxp3, goat anti-RUNX3 or anti granzyme B (anti-GZMB) (Santa Cruz), were applied according to our previous published method [31], [32].

The samples, processed for multiple fluorescence (DAPI, FITC, Cy3 and CF^TM^647), were sequentially excited with the 405 nm/25 mW lines of a blue diode laser, the 488-nm/20 mW lines of the Argon laser, the 543 nm/1.2 mW lines of a HeNe laser and the 633 nm/102 mW lines of a HeNe laser.

Optical sections were obtained at increments of 0.3 µm in the z-axis and were digitized with a scanning mode format of 512 x 512 or 1024 x 1024 pixels and 256 grey levels. For colocalization analyses, the different channel images were acquired independently, and photomultiplier gain for each channel was adjusted to minimize background noise and saturated pixels. Once acquired, images were not modified further.

The degree of colocalization between red (Cy3) and green signal (FITC) was calculated based on the integrated density of each signal independently for each different sections. Manders coefficient was calculated with ImageJ using the JACoP tool [34]. Manders coefficient varies from 0 to 1, corresponding to non-overlapping images and 100% colocalisation between the two images, respectively. The intensity of fluorescence for each marker was multiplied for the corresponding Manders coefficient, in order to obtain the normalized colocalization levels.

To evaluate triple colocalization of FITC, Cy3 and CF^TM^64, data were analyzed with ImageJ by selecting single slices and the co-localization of three different molecules was evaluated by the presence, intensity and distribution of the color resulting from the overlapping of RUNX3, CD8 and ThPOK, and the color resulting from the overlapping of RUNX3 and CD8 [35]. In cases where we had three fluorochromes, the triple colocalization was regarded as being separate and not contributing to the double.

### Statistical Analysis

All quantitative data for NM, MA, and CRC are reported as mean ± SE. The difference in average expression in the different groups of colorectal lesions, was tested for statistical significance using Kruskal-Wallis analysis, followed by Student-Newman-Keuls tests. The value of P<0.05 was chosen to indicate a significant difference.

## Results

### Western Blot Analyses of CD4, CD8, and CD56

**Figure 1:**
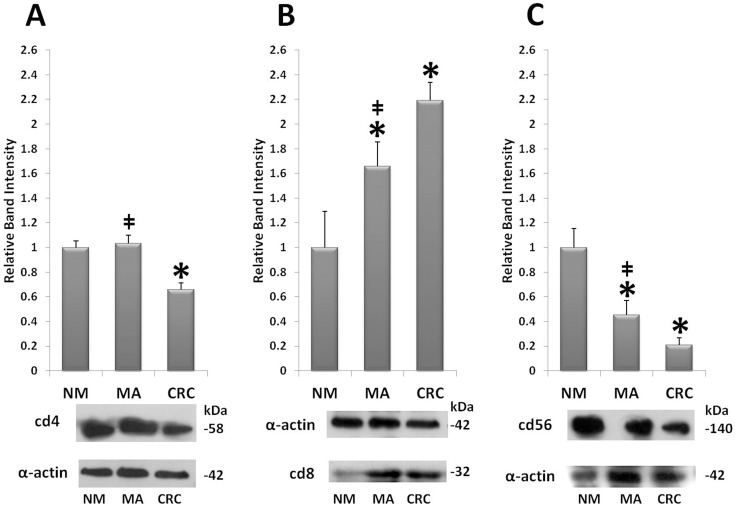
Quantification of lymphocytes subpopulations markers. Western blot analysis of normal colorectal mucosa (NM), microadenomas (MA), and colorectal cancer (CRC), using anti-CD4, anti-CD8, anti-CD56 antibodies. Mean densitometric data of protein expression were analyzed using a NIH Image J software. Band intensities for NM were arbitrarily set to 1. *P<0,05 vs NM; ‡ P<0,05 vs CRC. **Panel A:** CD4 densitometric analysis, and specific bands at 58 kD for CD4 and at 42 kD for α-actin. **Panel B:** CD8 densitometric analysis and bands at 32 kD for CD8 and 42 kD for α-actin. **Panel C:** CD56 densitometric analysis, and bands at 140 kD for CD56 and at 42 kD for α-actin.

In order to investigate the expression profile of lymphocyte subpopulations involved in colorectal carcinogenesis and affected by ThPOK, we evaluated a panel of antibodies specific for proteins which identify CD4+, CD8+ and CD56+ lymphocytes. Lysates of NM, MA, and CRC were analyzed by Western blotting followed by densitometric analysis of the immunoreactive bands. Western blotting in analyzing the protein profile of CD4 showed one specific immunoreactive band at 58 kD. CD4 protein levels in MA were not significantly increased with respect to NM (Figure 1, panel A; densitometric ratio 1.03±0.07), whereas decreased levels were observed in CRC. (Figure 1, panel A; densitometric ratio of 0.65±0.05, p<0.05 vs NM). Western blotting data showed that the levels of CD8 protein had a significant upward increase from NM to CRC, with a slightly detectable band in NM; densitometric ratios were 1.66±0.20 for MA and 2.19±0.15 for CRC. (Figure 1, panel B; band at 32 kD). The CD56 protein levels, corresponding to a 140-kD band, decreased fivefold during colorectal cancer progression (Figure 1, panel C; densitometric ratios of 0.45±0.11 in MA and 0.20±0.05 in CRC versus NM).

### Quantification of ThPOK Protein and mRNA

**Figure 2:**
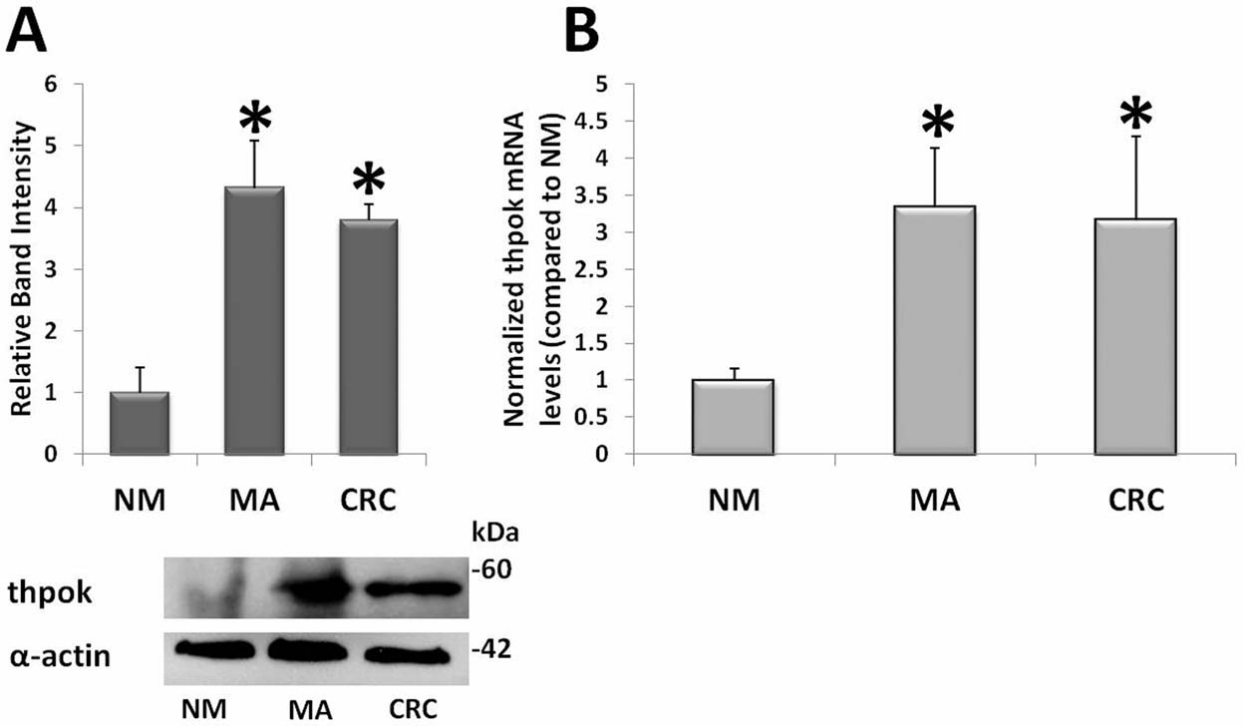
ThPOK protein and mRNA during colorectal neoplastic progression. Measurement of ThPOK protein and mRNA in normal colorectal mucosa (NM), microadenomas (MA), and colorectal cancer (CRC). **Panel A:** NM, MA and CRC were subjected to SDS–PAGE/western blot using the anti-ThPOK antibody; densitometric analysis and bands at 60 kD for ThPOK and at 42 kD for α-actin.* P<0.05 vs NM. **Panel B:** Real-time PCR analysis of ThPOK mRNA in NM, MA and CRC. *P<0,05 vs NM. Band intensity of ThPOK protein and ThPOK mRNA level for NM were arbitrarily set to 1.

The amounts of ThPOK protein and mRNA were quantified using Western blot and qRT-PCR analyses. Western blotting showed that the expression of ThPOK protein was markedly enhanced during colorectal carcinogenesis. ThPOK protein levels were increased 4.3±0.77-folds in MA and 3.78±0.27-fold in CRC compared to NM (Figure 2, panel A). The amount of ThPOK mRNA confirmed an upregulation since the early neoplastic lesions, with a fold change of 3.33±0.79 in MA and 3.16±1.13 in CRC vs NM (Figure 2, panel B).

### Fluorescence Analysis of CD4+, CD8+, CD56+, and ThPOK+ cell Infiltration

In order to evaluate differences between NM, MA and CRC in a quantitative mode, we performed immunofluorescence experiments by confocal microscopy which allows not only to obtain a good resolution of subcellular structures in very thick samples but also to perform the quantification of the proteins detected. The confocal analysis confirmed the data obtained by Western blot analysis.

**Figure 3:**
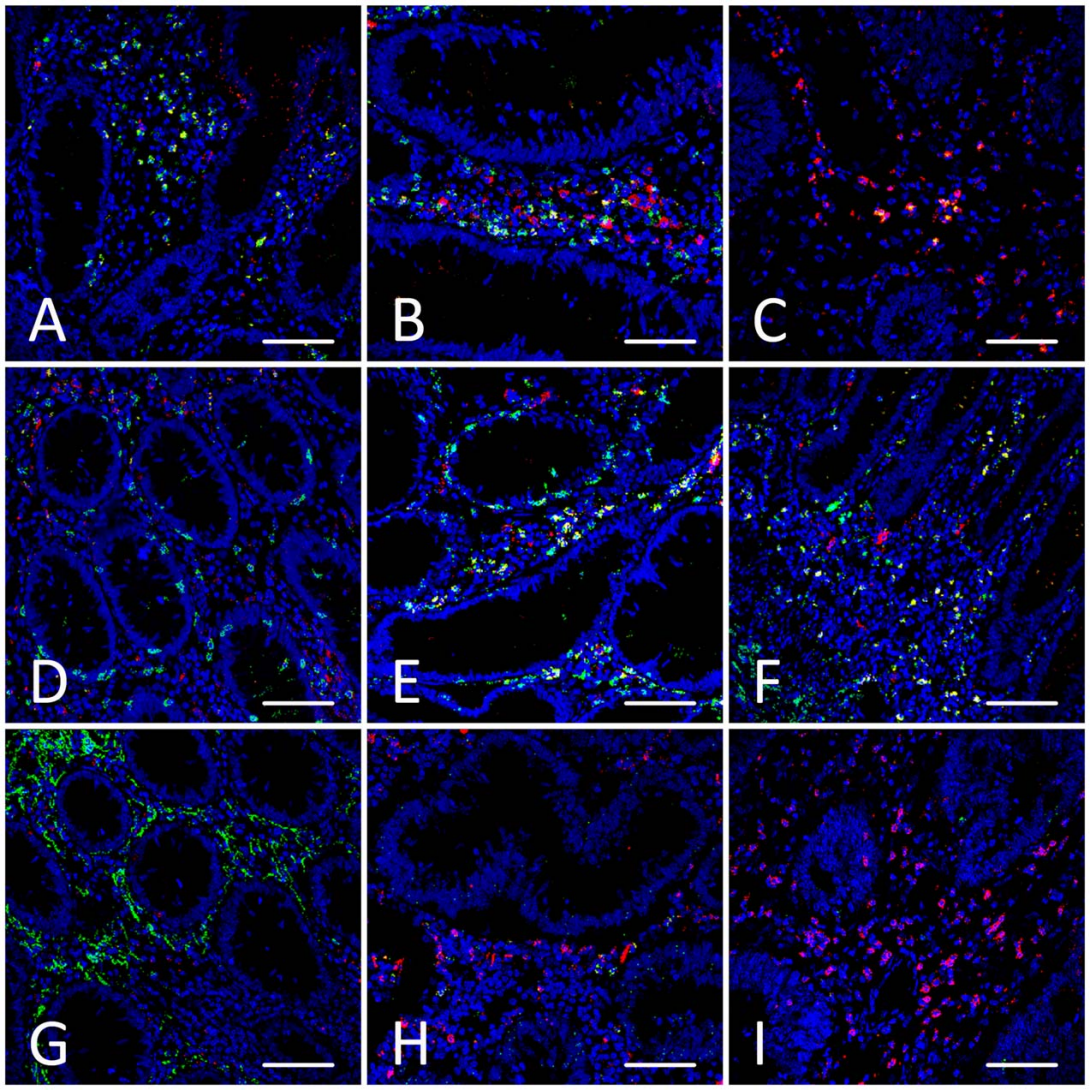
Confocal immunofluorescence staining. Examples of confocal analysis of cryosections of normal colorectal mucosa (NM), microadenoma (MA), and colorectal carcinoma (CRC), labelled by DAPI (blue), ThPOK (red), CD4 (green), CD8 (green), and CD56 (green). Double immunolabelled cells appear as yellow spots. **Panels A-C**: Colocalization imaging of ThPOK with CD4 in NM (panel A), MA (panel B) and CRC (panel C). **Panels D-F**: Double immunolabelling performed by ThPOK and CD8 in NM (panel D), MA (panel E) and CRC (panel F). **Panels G-I**: Immunostaining with ThPOK and CD56 in NM (panel G), MA (panel H) and CRC (panel I). Scale bar  = 80 µm.

The fluorescence level of CD4 immunostaining was similar in NM and in MA. Furthermore, there was a significant decrease of CD4 immunostaining in CRC compared to NM and MA (Table 1 and Figure 3, panels A-C).

**Table 1:** Immunofluorescence quantification by confocal analysis.

	CD4 IFIS (mean ± SEM)	CD8 IFIS (mean ± SEM)	CD56 IFIS (mean ± SEM)	ThPOK IFIS (mean ± SEM)
NM	26.61±3.26	17.22±2.64	63.94±11.98	24.9±3.0
MA	27.21±2.31	30.74±3.56*	24.32±5.18*	44.69±5.64*
CRC	13.35±2.59*	46.25±6.42*	8.06±3.31*	45.41±5.02*

Fluorescence quantification (ImmunoFluorescence Intensity Score, IFIS, see Materials and Methods) of CD4, CD8, CD56 and ThPOK in normal colorectal mucosa (NM), microadenoma (MA) and colorectal carcinoma (CRC). * p<0.05 vs normal colorectal mucosa.

Confocal analysis of CD8+ cells showed that the mean IFIS increased steadily from NM to MA and CRC (Table 1, P<0.05 between all pairs of groups, and Figure 3, panels D-F,).

The expression of CD56 was very high in all samples of NM, decreasing substantially in MA, and showing the lowest level of expression in CRC; in fact, very few stained cells were present in most samples of CRC (Table 1, P<0.05 between all pairs of groups, and Figure 3, panels G-I).

The samples of NM were weakly stained for ThPOK, whereas both in MA and in CRC ThPOK staining was significantly higher (Table 1, P<0.05 vs NM, and Figure 3 panels A-I).

### Colocalization Analyses

**Figure 4:**
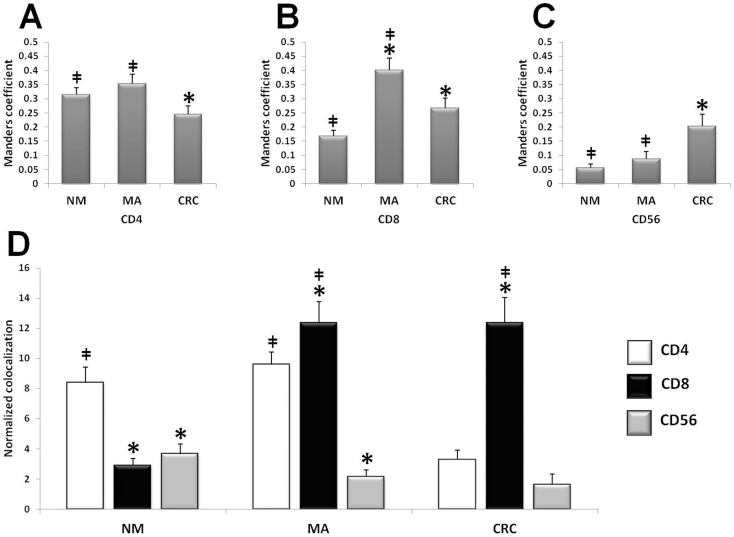
Colocalization analysis. Quantitative analysis of co-expression levels by Manders coefficient in normal mucosa (NM), microadenomas (MA), and colorectal cancer (CRC), analyzing the ratio between ThPOK/CD4 (panel A), ThPOK/CD8 (panel B), and ThPOK/CD56 (panelC). *P<0,05 vs NM; ‡ P<0,05 vs CRC. Panel D: Normalized co-expression levels in normal mucosa (NM), microadenomas (MA), and colorectal cancer (CRC), between ThPOK and CD4 (white bars), ThPOK and CD8 (black bars), and ThPOK and CD56 (gray bars). *P<0,05 vs CD4; ‡ P<0,05 vs CD56.

In order to identify the type of stromal cells positive for CD4, CD8 and CD56 which mostly expressed ThPOK, we performed cellular colocalization studies by double immunofluorescence analysis coupled with confocal microscopy. Figure 4 shows the localization of anti-CD4, anti-CD8 and anti-CD56 antibodies coupled with ThPOK staining in samples of NM, MA, and CRC. The colocalization image was used to calculate the overlap coefficient according to Manders [32]. We observed interesting changes in the colocalization levels during neoplastic progression (Figure 4).

The degree of ThPOK/CD4 colocalization in NM and MA was similar, it was significantly lower in colorectal carcinomas (Figure 4, panel A). The Manders coefficient for ThPOK and CD8 showed a peak in MA samples. Although the increased alignment of ThPOK and CD8 was more prominent in MA compared to colorectal carcinomas, in both groups it was statistically higher than in NM (P<0.05 between all pair of groups, Figure 4, panel B). The colocalization degree of ThPOK with CD56 increased in samples of colorectal carcinomas compared to normal mucosa and microadenomas (P<0.05 Figure 4, panel C), although the level of CD56 in carcinomas was very low.

By normalizing the co-expression data to the fluorescence levels, we observed that, in NM, most of ThPOK+ cells was CD4+, and a lower level of colocalization was observed between ThPOK and the others markers. In MA the level of colocalization of ThPOK with CD4 remained similar to NM, whereas the highest colocalization was with CD8, statistically higher when compared to NM (P<0.05 vs NM), and smaller colocalization with CD56 (P<0.05 vs NM). In CRC there was a lower level of colocalization with CD4 (P<0.05 vs both NM and MA), the increased levels of double staining of ThPOK and CD8 was similar to MA, and the amount of ThPOK+/CD56+ cells was almost undetectable (Figure 4, panel D).

### ThPOK and Treg Lymphocytes

**Figure 5:**
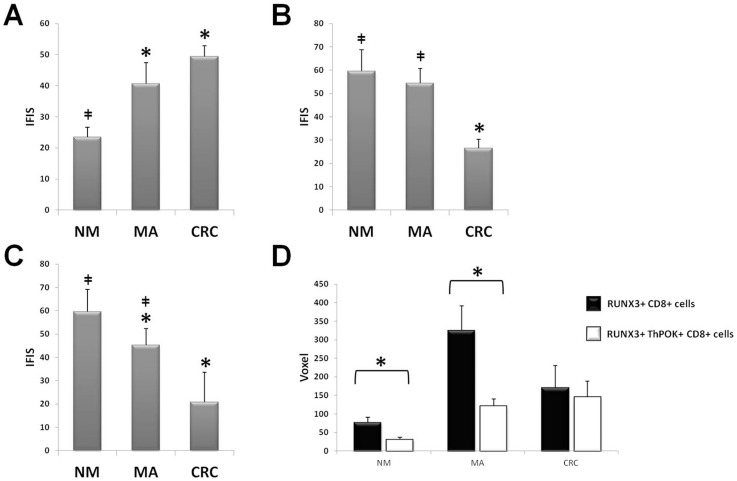
Foxp3, GZMB and RUNX3 fluorescence levels. Fluorescence levels of Foxp3 (panel A), GZMB (panel B), RUNX3 (panel C) immunostaining. *P<0,05 vs NM; ‡ P<0,05 vs CRC. RUNX3 level in CD8+ T cells coexpressing (white bars) and not coexpressing (black bars) ThPOK in NM, MA and CRC (panel D). *P<0,05.

We performed cellular colocalization studies by triple immunofluorescence analysis coupled with confocal microscopy in order to look for a coexpression of ThPOK and Foxp3 in colorectal carcinogenesis. ThPOK did not colocalize with Foxp3 in all the specimens, but either shown a comparable expression profile. The IFIS levels of Foxp3 increased from NM (IFIS 23.5±3.2) to MA (IFIS 40.7±6.7) and CRC (IFIS 49.4±3.4) (Figure 5, panel A).

### ThPOK and CD8+ Effector Functions

We subsequently analyzed the presence of effector markers, as GZMB or RUNX3, in CD8+ cells regarding to the ThPOK presence, by performing triple immunofluorescence staining.

The coexpression of ThPOK and GZMB in CD8+ cells wass almost undetectable; ThPOK did not colocalize with GZMB, neither in NM, MA or CRC. The amount of GZMB decreased from NM (IFIS 59.6±9.1) to CRC (IFIS 26.6±3.7), in contrast to the increase of ThPOK since microadenomas (Figure 5, panel B).

Also the levels of RUNX3 fluorescence decreased from NM (IFIS 59.6±9.6) to MA (IFIS 45.3±6.9) and to CRC (IFIS 20.8±12.2) (Figure 5, panel C).

**Figure 6:**
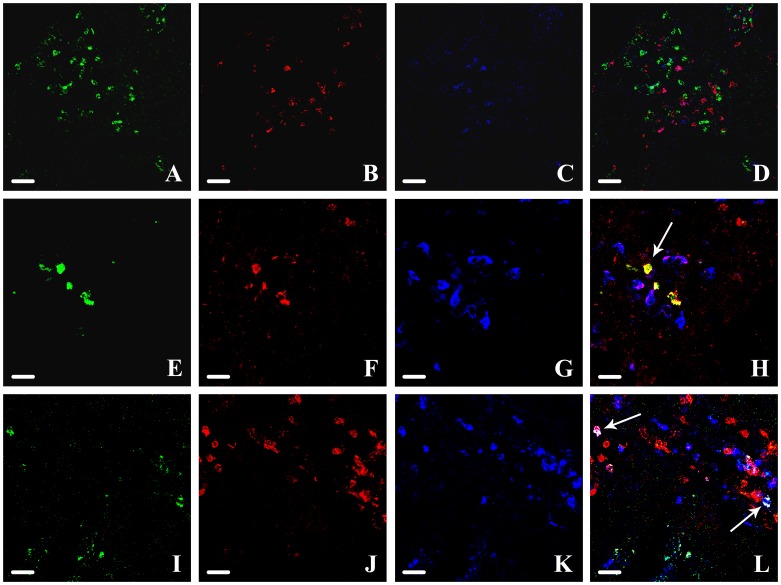
RUNX3, CD8 and ThPOK triple fluorescence. Triple colocalization of RUNX3, CD8 and ThPOK in NM (panel A-D, Scale bar = 50 µm), MA (panel E-H, Scale bar = 30 µm) and CRC (panel I-L, Scale bar = 30 µm). RUNX3: green (panel A, E, I); CD8: red (panel B, F, J); ThPOK: blue (panel C, G, K). Merge (panel D, H, L): CD8+ cells expressing RUNX3: yellow (arrow in panel H); CD8+ cells coexpressing RUNX3 and ThPOK: white (arrows in panel L).

In all the samples the levels of RUNX3-ThPOK-coexpressing CD8+ T cells were lower with respect to the levels of RUNX3 - positive CD8+ T cells. This was more evident in MA, where there was a maximum level of RUNX3-positive CD8+ T cells. This difference was not significant in CRC samples, where the level of RUNX3-ThPOK-coexpressing CD8+ T cells and of RUNX3-positive CD8+ T cells became equal (Figure 5, panel D and Figure 6).

## Discussion

Our study focused on colorectal carcinogenesis, and expecially on the very early stages of colorectal cancer progression, identified by dysplastic aberrant crypt foci, also referred to as microadenomas [30], [36]. In this context we tried to define a possible regulator of the transformations making the immune system unable to control the development of colorectal cancer at the very early stages of onset.

We analyzed helper T lymphocytes, cytotoxic T lymphocytes, and natural killer T cells, identified respectively by CD4, CD8 and CD56 markers in human normal colorectal mucosa, microadenomas and carcinomas, using immunofluorescence techniques and protein quantification analyses by Western blot. In microadenomas no significant change in CD4+ cells was observed with respect to normal mucosa. On the other hand, a significant decrease of these cells in carcinomas was observed. Moreover, we noted a gradual increase of CD8+ T cells, during tumour progression. Finally a strong decrease of CD56+ cells in microadenomas was apparent, and this decrease was even more pronounced in carcinomas, where CD56+ cells were almost undetectable.

We then analyzed ThPOK, a protein with a prominent role in the commitment of some leucocytic lineages, such as helper, cytotoxic and natural killer T cells, which have a pivotal role in defining the aggressiveness and prognosis of various types of cancer, including colorectal carcinomas [4], [5]. ThPOK was observed to have an unexpected increase in preneoplastic colorectal lesions, suggesting its involvement since the earliest phases of immune system remodelling in the colorectal neoplastic microenvironment.

By morphological analysis with immunofluorescence coupled with confocal microscopy, we observed changes in the immunological pattern during neoplastic progression. In normal mucosa there was a predominance of CD56+ cells, and a lower infiltration of T lymphocytes (both helper and cytotoxic), associated with lower levels of ThPOK. In microadenomas, we observed both a decrease of CD56+ cells and an increase of CD8+ T cells, paralleled by a relevant increase of ThPOK immunostaining. Finally, in carcinomas, the presence of CD56+ cells was scarce, with a prevalence of CD8+ T cells together with an increase of ThPOK labeling.

Many studies have analyzed the presence/amount of helper and cytotoxic T cells in colorectal carcinomas, but only few evaluated changes during colorectal cancer development, since normal mucosa and microadenomas. In addition, data on quantification of lymphocyte populations in colorectal carcinogenesis are currently elusive.

It has been demonstrated that the increased expression of genes specific for cytotoxic T lymphocytes, as CD8α, granzyme B, or perforin was related to the absence of early metastatic invasion of colorectal cancer, and it could also improve patient survival [37], [38].

At the moment, data are not available regarding a tumour-specific activation of CD8+ T cells, but it has been demonstrated a tumour-induced inhibition of CD8+ T cells, related to tumour stage. There are at least three mechanisms to account for a disfunction of CD8+ cells: *i*) cytotoxic T lymphocytes may be inactive, as revealed by the low levels of cytotoxic markers, *ii*) cytotoxic T lymphocytes may be apoptotic, *iii*) cytotoxic T lymphocytes may be immature [39]–[44].

ThPOK was initially considered a regulator of CD4+ lineage. Further experiments have shown its activation not only in CD4+ lymphocytes, but also in peripheral CD8+ cells [45]. These data are consistent with the hypothesis that ThPOK is important in maintaining the CD4+ phenotype in physiological conditions, as in normal mucosa this protein is mostly expressed in CD4+ cells.

Foxp3 is a master regulator of a class of immunosuppressive T cell, that has a central role in cancer progression. Our studies failed to find a correlation between foxp3 and ThPOK, but as reported by others works [46], [47], an increase of foxp3+ cells during colorectal cancer progression was evident.

However, the novelty as well as the main finding of the present work is the observation that ThPOK becomes prevalent in CD8+ T cells during the earliest dysplastic phases of colorectal cancer development. This fact outlines a new perspective on the hypothesis of the effectiveness of CD8+ T cells against colorectal cancer cells, suggesting a possible mechanism of the reduced immune response to colorectal tumours.

Up to now, no study has investigated the role of ThPOK, or other protein regulators of immune plasticity, in peripheral organs during progression of solid tumours. ThPOK is required both for CD4+ cells commitment and for helper identity maintenance. ThPOK, when introduced into mature CD8+ T cells, prevents the inappropriate expression of CD8-lineage genes, including CD8, perforin and granzyme B, and the transcription factors Runx3 and Eomes [28]. Thus, ThPOK could represent a new mechanism explaining the low effector property of CD8+ T cells against tumour cells, just mediated by ThPOK. Since ThPOK controls and decreases various cytotoxic effectors, it could be responsible for the inactivation of CD8+ T cells and, thus, it could be actively involved in contributing to the “immune escape” phenomenon.

This can be supported also by our results that indicate a decrease of GZMB expression during colorectal neoplastic progression in parallel with an increase level of ThPOK amount. Furthermore, ThPOK expression in CD8+ cell seems to exclude GZMB expression, in NM as well as in MA and CRC.

RUNX3 levels decreased during colorectal carcinogenesis, too, as already reported by others studies [48]. By colocalization analysis we observed that CD8+ cells expressing RUNX3 decreased in carcinomas, while the presence of ThPOK increase in the same cells. This may suggest a role of ThPOK in the modification of CD8+ cells activity against cancer cells. Functional studies are necessary to clarify a cause-effect mechanism for this observation.

These observations, considered together, seems to suggest putative roles of ThPOK not only in maintaining the phenotype of helper T lymphocytes, as already demonstrated by other studies [29], but also in controlling the inactivation of CD8+ T cells.

Many works, even recently, have analyzed the presence of cytotoxic or helper T cells in the tumour microenvironment of colorectal cancer [49]. NK and NKT cells have a key role in normal homeostasis and tissue differentiation of the gut [17], [18]; in vitro, they act quite well as effector cells against tumour target cells [50]. The role of NK and NKT cells infiltration in colorectal cancer is not well understood [19], and few studies have analyzed CD56+ cells in colorectal cancer progression: they reported low levels of CD56+ cells in colorectal cancer [22], [23], and the evidence that infiltration of NK cells in malignant tumours was associated with a favourable outcome [21]. The decrease in number of CD56+ cells during neoplastic progression foreshadows a major role for natural killer cells in controlling tumour progression.

ThPOK is considered, among other roles, a regulator of the functions of natural killer T cells [51]. Our study shows its lack of influence on natural killer and natural killer T cells, identified by the CD56 marker, during colorectal cancer progression. We did not find a consistent presence of ThPOK-CD56 colocalization in normal mucosa, and the number of CD56+ cells became almost undetectable during neoplastic progression. However, the marked decrease of CD56+ cells, together with the action exerted by ThPOK in CD8+ T lymphocytes, may be the key mechanisms of tumour microenvironment modification, referred as immunoediting, which makes the immune system inefficient against neoplastic growth.

The number of blood white cells which have been typed is currently growing. Recent studies performed by flow cytometry showed a great plasticity of the immune system in terms of patterns or networks assumed by various leucocytic lineages. The results of the present study suggest that a pattern of proteins might exist which could define an overall status of the immune system, not a subpopulation of leukocytes in particular. In other words, colorectal cancer development could somehow influence not only the type of infiltrating cells themselves, but also drive its plasticity. ThPOK may be considered one of the main regulators of such plasticity, influencing the immune escape mechanisms since the early onset of neoplastic clones.
